# Correction to “Biomechanical Advantages of Novel Duet Screws Plus Bilateral Satellite Rods Fixation in the Correction Surgery for Adult Spinal Deformity”

**DOI:** 10.1111/os.70275

**Published:** 2026-03-03

Z. He , Y. Chen , Z. Liu , et al., “Biomechanical Advantages of Novel Duet Screws Plus Bilateral Satellite Rods Fixation in the Correction Surgery for Adult Spinal Deformity,” Orthopaedic Surgery 17 (2025): 2454–2466. 10.1111/os.70121.40667831 PMC12318676

In the originally published version of this article, the authors’ affiliation was incorrect.

The published address read:

“Department of Spine Surgery, Affiliated Drum Tower Hospital, Medical School of Nanjing University, Nanjing, China”

The correct affiliation should be:

“Division of Spine Surgery, Department of Orthopedic Surgery, Nanjing Drum Tower Hospital, Affiliated Hospital of Medical School, Nanjing University, Nanjing, China.”

We apologize for this error.

